# m^6^A-modified *Mid1* promotes sevoflurane-induced cognitive impairment in neonatal mice by ubiquitin-mediated degradation of Syngap1

**DOI:** 10.1038/s12276-026-01747-7

**Published:** 2026-06-05

**Authors:** Jian-Jun Shen, Ying-Jie Yang, Ying-Ying Tang, Shuo Yan, Mei-dan Ying, Wen-Hu Chen, Li-Li Xu

**Affiliations:** 1 Department of Anesthesia, the Second Affiliated Hospital, Zhejiang University School of Medicine, Hangzhou, Zhejiang Province China; 2 Department of Anesthesia, Women’s Hospital, Zhejiang University School of Medicine, Hangzhou, Zhejiang Province China; 3 Nanhu Brain–Computer Interface Institute, Hangzhou, Zhejiang Province China; 4 Institute of Pharmacology & Toxicology, College of Pharmaceutical Sciences, Zhejiang University, Hangzhou, Zhejiang Province China; 5 Department of Biochemistry and Molecular Biology, School of Basic Medical Sciences & Forensic Medicine, Hangzhou Medical college, Hangzhou, Zhejiang Province China

**Keywords:** Drug development, Health care, Development of the nervous system

## Abstract

Investigating the cognitive effects of sevoflurane exposure during early development is essential due to its potential long-term neurodevelopmental impacts. This investigation systematically explored the molecular basis of sevoflurane-induced cognitive impairment, with emphasis on m^6^A RNA modifications and ubiquitin-dependent proteostasis involving *Mid1* and Syngap1. Using integrated approaches, including methylated RNA immunoprecipitation sequencing (MeRIP-seq), transcriptomic profiling, neurobehavioural testing and molecular analyses, 2091 m^6^A methylation sites were identified that were differentially regulated. Mechanistically, *Mid1* was found to orchestrate Syngap1 degradation via the ubiquitin–proteasome pathway, establishing a direct link between protein stability control and cognitive outcomes. Behavioural phenotyping demonstrated that *Mid1* suppression ameliorated learning and memory deficits in sevoflurane-exposed mice, which was corroborated by improved neuronal viability and attenuated apoptotic signalling in biochemical assays. Epigenetic regulation studies further revealed that the m^6^A eraser ALKBH5 and the reader YTHDF2 collaboratively modulate *Mid1* mRNA stability, thereby contributing to neuropathological progression. Pathway analysis uncovered *Mid1*–Syngap1 axis-mediated dysregulation of MAPK signalling cascades, proposing this network as a potential therapeutic target. Collectively, the present findings delineated a novel m^6^A-ubiquitin regulatory circuit centred on *Mid1* that drives sevoflurane-associated cognitive dysfunction, offering mechanistic insights for the development of neuroprotective interventions against anaesthesia-related neurotoxicity in paediatric and other at-risk populations.

## Mid1 and Syngap1 regulate neurotoxicity from sevoflurane

Sevoflurane, a commonly used anaesthetic, has been linked to potential cognitive deficits when administered during critical neurodevelopmental periods. This study explores the molecular mechanisms underlying these effects, focusing on m^6^A RNA methylation and its impact on gene expression in neonatal mice. Researchers identified 2091 differentially methylated m^6^A peaks in hippocampal tissues following sevoflurane exposure, with significant upregulation of the *Mid1* gene, an E3 ubiquitin ligase. Functional studies showed that *Mid1* knockdown improved cognitive performance and reduced neuronal apoptosis, implicating *Mid1* in sevoflurane-induced neurotoxicity. The study further revealed that *Mid1* regulates Syngap1, a synaptic plasticity protein, through ubiquitination, affecting the MAPK signalling pathway. These findings highlight the role of m^6^A modifications and *Mid1*–Syngap1 signalling in anaesthetic-induced cognitive dysfunction, suggesting potential therapeutic targets for mitigating neurotoxic effects. Future research could focus on developing interventions to protect cognitive function during early-life anaesthetic exposure.

This summary was initially drafted using artificial intelligence, then revised and fact-checked by the author.

## Introduction

Sevoflurane is widely used as an anaesthetic in both paediatric and adult populations due to its favourable pharmacokinetic properties, including rapid onset and recovery. Although generally considered safe, accumulating preclinical evidence suggests that sevoflurane exposure during critical neurodevelopmental periods may induce long-term cognitive deficits and behavioural abnormalities^1^. Rodent studies have shown that neonatal exposure during synaptogenesis (postnatal days 7–10) reduces synaptic density in hippocampal cornu ammonis 1 (CA1) neurons through disruption of dendritic arborization^2,3^. Previous work has corroborated these findings, demonstrating dose-dependent impairments in cognitive performance, emotional regulation and social behaviours following prolonged sevoflurane exposure (≥3 h)^4,5^. These observations underscore the necessity to elucidate the molecular mechanisms underlying sevoflurane-induced neurotoxicity.

The regulatory role of N^6^-methyladenosine (m^6^A) methylation in neural development and inflammatory responses has garnered increasing attention^6–8^. Dysregulation of m^6^A modification has been implicated in neurodevelopmental disorders through mechanisms involving altered neural stem cell differentiation and synaptic protein synthesis^9,10^. In the experimental models used herein, sevoflurane exposure induced 2,091 differentially methylated m^6^A peaks in hippocampal tissues, with predominant localization in mRNA coding sequences (CDS) and 3’ untranslated regions (3’UTR). This spatial distribution pattern is consistent with established mechanisms wherein CDS-located m^6^A modifications modulate translational efficiency, while 3’UTR modifications influence mRNA stability.

Through integrated analysis of m^6^A sequencing and RNA sequencing (RNA-seq) datasets, *Mid1* emerged as a key candidate gene showing significant upregulation post-sevoflurane exposure. *Mid1* encodes an E3 ubiquitin ligase associated with neuronal migration defects in X-linked Opitz G/BBB syndrome^11,12^. Functional studies revealed that *Mid1* knockdown attenuated sevoflurane-induced cognitive deficits, as quantified by improved performance in spatial navigation and associative memory tasks^13^. Histopathological assessments further demonstrated reduced neuronal apoptosis following *Mid1* inhibition. These findings align with recent reports linking *Mid1* to Tau phosphorylation regulation via protein phosphatase 2A (PP2A) interaction in neurodegenerative models.

Mechanistic investigations identified Syngap1, a synaptic plasticity regulator associated with intellectual disability, as a downstream effector of *Mid1*. Biochemical analyses suggest that *Mid1* modulates Syngap1 ubiquitination, thereby influencing mitogen-activated protein kinase (MAPK) signalling pathways essential for neuronal survival^14,15^. This interaction gains particular significance given the established role of MAPK–extracellular signal-regulated kinase (ERK) signalling in activity-dependent protein synthesis during synaptic plasticity^16,17^. In vivo validation studies confirmed that combinatorial targeting of *Mid1* and Syngap1 effectively reversed sevoflurane-induced cognitive impairments^18^.

Collectively, these findings establish a mechanistic link between m^6^A methylation dynamics and *Mid1*–Syngap1 signalling in anaesthetic-induced neurotoxicity. The identified pathways provide molecular insights into clinical observations of increased neurodevelopmental risks associated with multiple early-life anaesthetic exposures. This work advances our understanding of anaesthesia-related cognitive dysfunction and highlights potential therapeutic targets for mitigating neurotoxic effects.

## Materials and methods

### Ethics statement

All experiments were approved by the Zhejiang University Institutional Review Board of Animal Studies (ZJU20210174) and were performed in accordance with the Guidelines and Recommendations for Experimental Animal Care and Use of the Ministry of Science and Technology of China^19^.

### Animal model and treatment

This study utilized P2 neonatal C57BL/6 mice (Shanghai SLAC Laboratory Animal) due to their favourable neurodevelopmental characteristics. The mice were randomly assigned to either the control (CON) group or the sevoflurane (Sevo) group. On postnatal days 6, 8 and 10, the neonatal C57BL/6 mice were placed in an anaesthesia chamber and exposed to a mixture of 3% sevoflurane and 60% oxygen (balanced with nitrogen) for 2 h. The CON group received the same gas mixture without sevoflurane. This exposure protocol aimed to simulate the clinical conditions of anaesthetic exposure, allowing for the assessment of its cognitive effects in a controlled environment.

### Methylated RNA immunoprecipitation sequencing

Following treatment, hippocampal tissues were harvested from both groups for methylated RNA immunoprecipitation sequencing (MeRIP-seq) analysis. Total RNA was extracted using TRIzol reagent (Thermo Fisher), following the manufacturer’s protocol to ensure high-quality RNA suitable for downstream applications. The MeRIP procedure involved the use of an anti-m^6^A antibody (CST) to specifically enrich for methylated RNA species. After immunoprecipitation, the enriched RNA was purified, and library preparation was performed to create sequencing libraries. These libraries were subsequently sequenced on a high-throughput sequencing platform, enabling the identification of m^6^A peaks across the transcriptome.

### RNA sequencing

Total RNA extracted from hippocampal tissues was utilized to construct sequencing libraries. The libraries were prepared according to established protocols, ensuring optimal representation of the transcriptome. Following library preparation, sequencing was conducted using a high-throughput platform, generating comprehensive data on gene expression profiles. The resulting raw sequencing data were subjected to rigorous bioinformatics analysis to identify differentially expressed genes and to analyse the distribution of m^6^A peaks.

### Quantitative reverse transcription PCR

Quantitative reverse transcription PCR (RT-qPCR) was employed to assess the expression levels of target genes, including *Mid1* and *Syngap1*. Total RNA was extracted from hippocampal tissues or cultured neurons and reverse transcribed to cDNA using a reverse transcriptase kit (TaKaRa). Specific primers were designed for each gene of interest, and RT-qPCR was performed using a TB Green mix (TaKaRa). The relative expression levels were calculated using the ΔΔCt method, normalizing against housekeeping genes such as ACTB.

### Western blot analysis

Protein extracts were prepared from hippocampal tissues or cultured neurons using lysis buffer (Thermo Fisher) containing protease inhibitors. The protein concentration was determined using a bicinchoninic acid (BCA) assay (Thermo Fisher). Proteins were separated by sodium dodecyl sulfate-polyacrylamide gel electrophoresis (SDS-PAGE) and transferred to polyvinylidene difluoride (PVDF) membranes (Millipore). The membranes were blocked with bovine serum albumin (BSA) or non-fat milk and probed with specific primary antibodies against Mid1 (Santa Cruz), Syngap1 (Abcam), Bax, Bcl-2, cleaved caspase 3, phosphorylated p38 (p-p38), phosphorylated ERK1/2 (p-ERK1/2), phosphorylated mitogen-activated protein kinase (p-MEK1/2) and glyceraldehyde-3-phosphate dehydrogenase (GAPDH) as a loading control (CST). After incubation with appropriate secondary antibodies, protein bands were visualized using chemiluminescent detection methods.

### siRNA transfection

To investigate the functional role of specific genes, primary neuronal cells were transfected with small interfering RNAs (siRNAs) targeting *Mid1*, *Alkbh5* or *YTHDF2* using Lipo2000 transfection (Thermo Fisher) reagent according to the manufacturer’s instructions. Non-targeting control siRNA was included as a baseline comparison. In addition, to explore the effects of gene overexpression, *Mid1* was introduced into neuronal cells using a plasmid construct containing the *Mid1* gene. This was also facilitated through a suitable transfection reagent, allowing for the investigation of the biological consequences of *Mid1* overexpression in the context of sevoflurane exposure.

### Plasmids transfection

Full-length cDNAs encoding human MID1, SYNGAP1, ubiquitin and the indicated truncation mutants were amplified by PCR and cloned into vectors bearing FLAG, HA, His, Myc or GFP tags. Cells were transfected using Lipofectamine® 3000 (Invitrogen/Thermo Fisher Scientific) according to the manufacturer’s instructions.

### Dual-luciferase reporter assay and mutagenesis

Fragments containing the wild-type or mutant *Mid1* 3′ UTR were inserted upstream of the psiCHECK-2 dual-luciferase vector. For reporter measurements, cells were co-transfected with the wild-type or mutant *Mid1* 3′ UTR constructs together with OE-ALKBH5, OE-YTHDF2 or the corresponding empty vector. At 24 h after transfection, firefly and Renilla luciferase activities were quantified using the Dual-Luciferase® Reporter Assay System (Promega) according to the manufacturer’s instructions.

### Behavioural assessments

Behavioural assessments were conducted to evaluate the cognitive effects of sevoflurane exposure. Behavioural assessments were conducted at postnatal days 31–36. The Morris water maze test was employed to assess spatial learning and memory. Mice were trained to locate a submerged platform within a pool of water, and latency to reach the platform was recorded. This test measures cognitive function by evaluating the ability of the mice to remember the location of the platform over repeated trials. In addition, the conditioned fear test was performed to assess memory and anxiety-related behaviours. Mice were subjected to a fear conditioning paradigm, and freezing behaviour was quantified as an indicator of memory retention. These behavioural assays provided critical insights into the cognitive impairments induced by sevoflurane exposure.

### Immunofluorescence detection

Histological analyses were performed to assess neuronal health and proliferation in the hippocampus. The terminal deoxynucleotidyl transferase dUTP nick-end labelling (TUNEL) (Thermo Fisher) assay was utilized to detect apoptotic cells within brain sections, allowing for the visualization of DNA fragmentation associated with apoptosis. This technique was crucial for quantifying the extent of neuronal loss following sevoflurane exposure. Furthermore, 5-bromo-2ʹ-deoxyuridine (BrdU) (Thermo Fisher) proliferation assays were conducted to evaluate cellular proliferation. Mice were injected with BrdU prior to tissue collection, and brain sections were processed for immunohistochemical detection of BrdU-positive cells. This analysis provided insights into the effects of sevoflurane on neurogenesis and cellular proliferation in the hippocampus. In addition, double staining for p-p38 and p-MEK1/2 was conducted to assess activation of the MAPK signalling pathway. Sections were incubated with specific antibodies against p-p38 and p-MEK1/2, followed by secondary antibodies conjugated to fluorescent dyes. Images were captured using a fluorescence microscope.

### Flow cytometry for apoptosis detection

Flow cytometry was employed to quantify apoptotic cells in neuronal cultures. Following treatment with sevoflurane and transfection with siRNAs, cells were harvested and stained with Annexin V-FITC and propidium iodide according to the manufacturer’s instructions (Thermo Fisher). The stained cells were analysed using a flow cytometer, allowing for the differentiation between live, early apoptotic, and late apoptotic or necrotic cells. This method provided quantitative assessment of the apoptotic response induced by sevoflurane exposure and the effects of gene knockdown.

### CHX treatment

To investigate the stability of the Syngap1 protein in the context of *Mid1* knockdown, cycloheximide (CHX; Seleckchem) was used to inhibit protein synthesis. Neuronal cells were treated with CHX at various time points following transfection with *Mid1* siRNA. Protein extracts were collected, and western blot analysis was performed to assess the levels of Syngap1 over time. This experiment enabled the determination of the half-life of Syngap1 and elucidated the role of *Mid1* in regulating its stability.

### RNA pull-down assay

To investigate the interaction between *Mid1* and YTHDF2, RNA pull-down assays were performed. Biotinylated RNA probes corresponding to the m^6^A-modified regions of *Mid1* mRNA were synthesized and incubated with neuronal cell lysates. Streptavidin beads were used to capture the RNA–protein complexes, which were then analysed by western blot analysis to detect the presence of YTHDF2. This assay provided insights into the binding dynamics between m^6^A-modified RNA and reader proteins, contributing to the understanding of mRNA regulation.

### Immunoprecipitation and co-immunoprecipitation

To investigate protein–protein interactions, immunoprecipitation and co-immunoprecipitation (Co-IP) assays were performed. Protein extracts from neuronal cells were incubated with specific antibodies against Mid1 or Syngap1, followed by the addition of protein A/G beads (Thermo Fisher) to capture the immune complexes. The precipitated proteins were eluted and analysed by western blot analysis to confirm the presence of target proteins. Co-IP assays were conducted in HEK293T cells co-transfected with Flag-Mid1 and Syngap1 plasmids to validate their interaction. The results from these assays provided valuable insights into the molecular mechanisms underlying the regulation of Syngap1 by *Mid1*.

### Lentiviral injection

To achieve stable knockdown of target genes in vivo, lentiviral vectors containing short hairpin RNA (shRNA) targeting *Mid1* and *Syngap1* constructs for *Mid1* were prepared. P2 neonatal C57BL/6 mice were subjected to intracranial injection of the lentiviral particles to ensure targeted delivery to the hippocampus. Following recovery, the mice were exposed to sevoflurane, and behavioural assessments were conducted to evaluate the cognitive effects of gene manipulation. This approach enabled examination of the long-term effects of *Mid1* modulation on cognitive function in the context of sevoflurane exposure.

### CCK8 assay

Cell viability was assessed using the Cell Counting Kit-8 (CCK8) assay (Dojindo). Primary neuronal cells were seeded in a 96-well plate and treated with sevoflurane or transfected with siRNAs. After the treatment period, CCK8 solution was added to each well, and the cells were incubated for 4 h. The absorbance was measured at 450 nm using a microplate reader.

### LDH-release assay

The lactate dehydrogenase (LDH) release assay (Dojindo) was performed to evaluate cell membrane integrity and cytotoxicity. Following treatment with sevoflurane and transfection with siRNAs, the culture medium was collected, and LDH levels were measured using a commercial LDH assay kit according to the manufacturer’s instructions. The amount of LDH released into the medium was quantified, providing an indication of cell death.

### Statistical analysis

For all experiments, data were collected and analysed using appropriate statistical methods. Experiments were performed in triplicate to ensure reproducibility, and differences between groups were assessed using Student’s t-test or one-way analysis of variance (ANOVA), with a significance threshold set at *P* <0.05. Statistical analyses were conducted using GraphPad Prism software, allowing for a comprehensive evaluation of the data and validation of the experimental hypotheses.

## Results

### Differential m^6^A modification profiles in hippocampal tissue following sevoflurane exposure

**Fig. 1:**
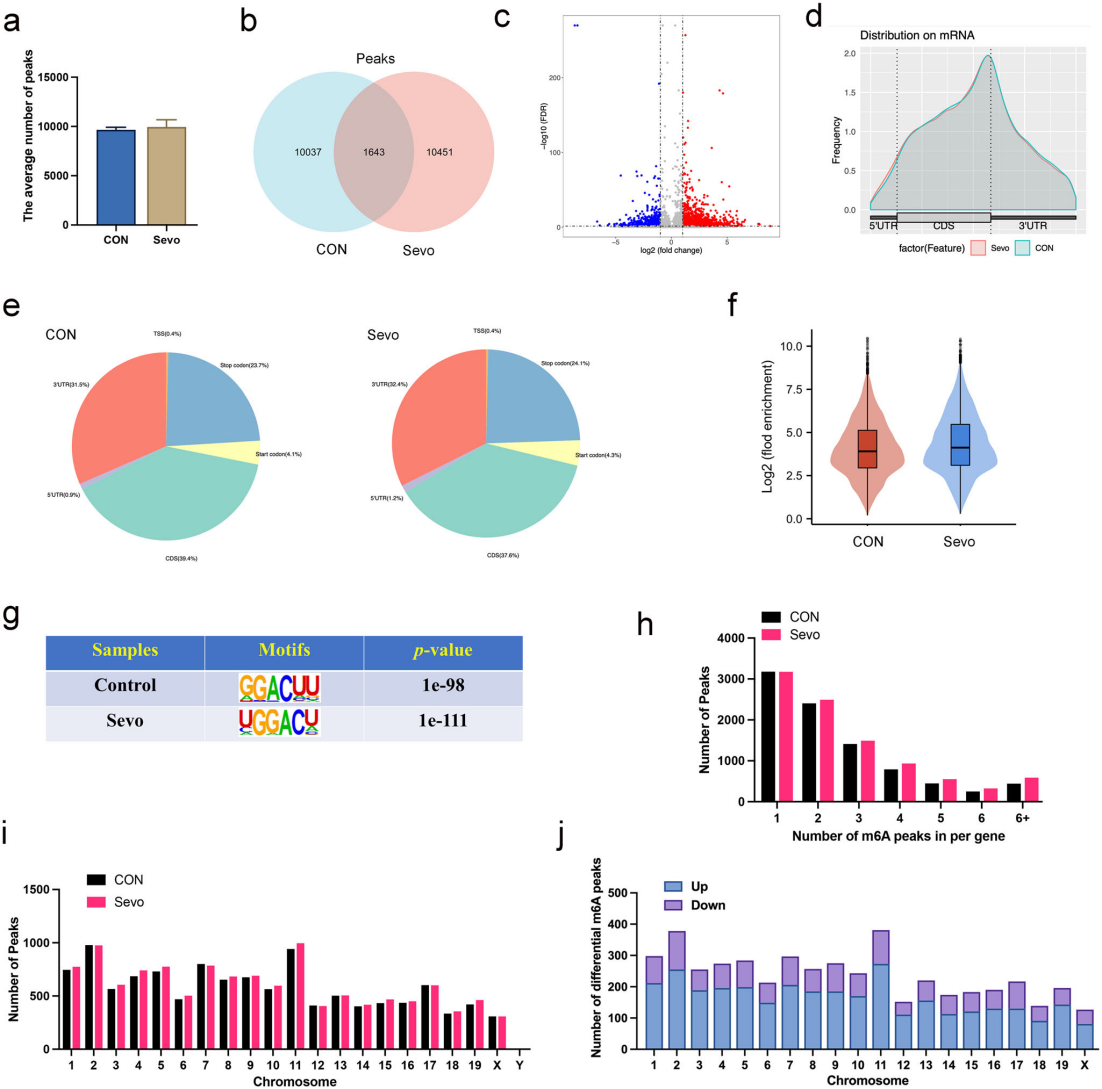
Differential m^6^A modification profiles in hippocampal tissue following sevoflurane exposure. Bar chart and Venn diagram showing the overlap of m^6^A peaks in the control (CON) and sevoflurane (Sevo) groups, with 1,643 peaks common to both groups (parts **a** and **b**). Volcano plot of differentially methylated m^6^A peaks, highlighting 1,543 hypermethylated and 548 hypomethylated peaks (fold change ≥1.0 and false discovery rate <0.05) (part **c**). Distribution of m^6^A peaks in genomic regions, including 3′ untranslated region (UTR), coding sequences (CDS) and stop codon regions, for both groups (parts **d** and **e**). Violin plot of average log2 enrichment ratios of m^6^A peaks in the CON (4.05) and Sevo (4.32) groups (part **f**). Sequence logo of the conserved m^6^A motif (GGACU) identified in the methylated RNA immunoprecipitation sequencing analysis (part **g**). Histogram showing the distribution of m^6^A peaks across individual mRNAs (part **h**). Histogram of m^6^A peak localization on human chromosomes, with enrichment on chr1, chr2, chr7 and chr11 (part **i**). The distribution of differentially methylated m^6^A peaks in human chromosomes (part **j**).

To investigate the biological role of m^6^A modifications in the cognitive functions of young mice subjected to multiple exposures with sevoflurane, MeRIP-seq was performed on hippocampal tissue samples from both the control (CON) and sevoflurane (Sevo) groups. This analysis identified an average of 11,680 m^6^A peaks in the CON group and 12,094 peaks in the Sevo group. Venn diagram analysis indicated that 1643 peaks were common to both groups (Fig. 1a,b). By comparing m^6^A methylation levels between the Sevo and CON groups, 2091 differentially methylated m^6^A peaks were identified in mRNA, of which 1543 were hypermethylated and 548 were hypomethylated (fold change ≥1.0 and false discovery rate <0.05) (Fig. 1c). Further analysis showed that m^6^A peaks preferentially localized near the CDS and 3′ UTR in both groups, with similar distribution patterns in gene regions. Specifically, the abundance of m^6^A peaks was notably higher in the 3′ UTR (31.5% in the CON group and 32.4% in the Sevo group), CDS (39.4% and 37.6%, respectively) and stop codon regions (23.7% and 24.1%, respectively) (Fig. 1d,e). In addition, m^6^A methylation enrichment analysis revealed that the average log2 enrichment ratio was 4.05 in the CON group and 4.32 in the Sevo group (Fig. 1f). Motif analysis characterized the m^6^A modification profile, identifying the most conserved motif among m^6^A peaks as GGACU (Fig. 1g). Analysis of the distribution of m^6^A peaks across individual mRNAs indicated that most mRNAs contained one or two m^6^A peaks (Fig. 1h). All m^6^A peaks were mapped to human chromosomes, particularly enriched on chr1, chr2, chr7 and chr11 (Fig. 1i), with dysregulated m^6^A peaks observed across all chromosomes but especially on chr1, chr2 and chr11 (Fig. 1j). This comprehensive profiling underscores the intricate role of m^6^A modifications in hippocampal tissue and their potential implications in cognitive function.

### Combined analysis of m^6^A sequencing and RNA-seq data reveals a strong correlation between *Mid1* and sevoflurane-induced cognitive impairment

**Fig. 2:**
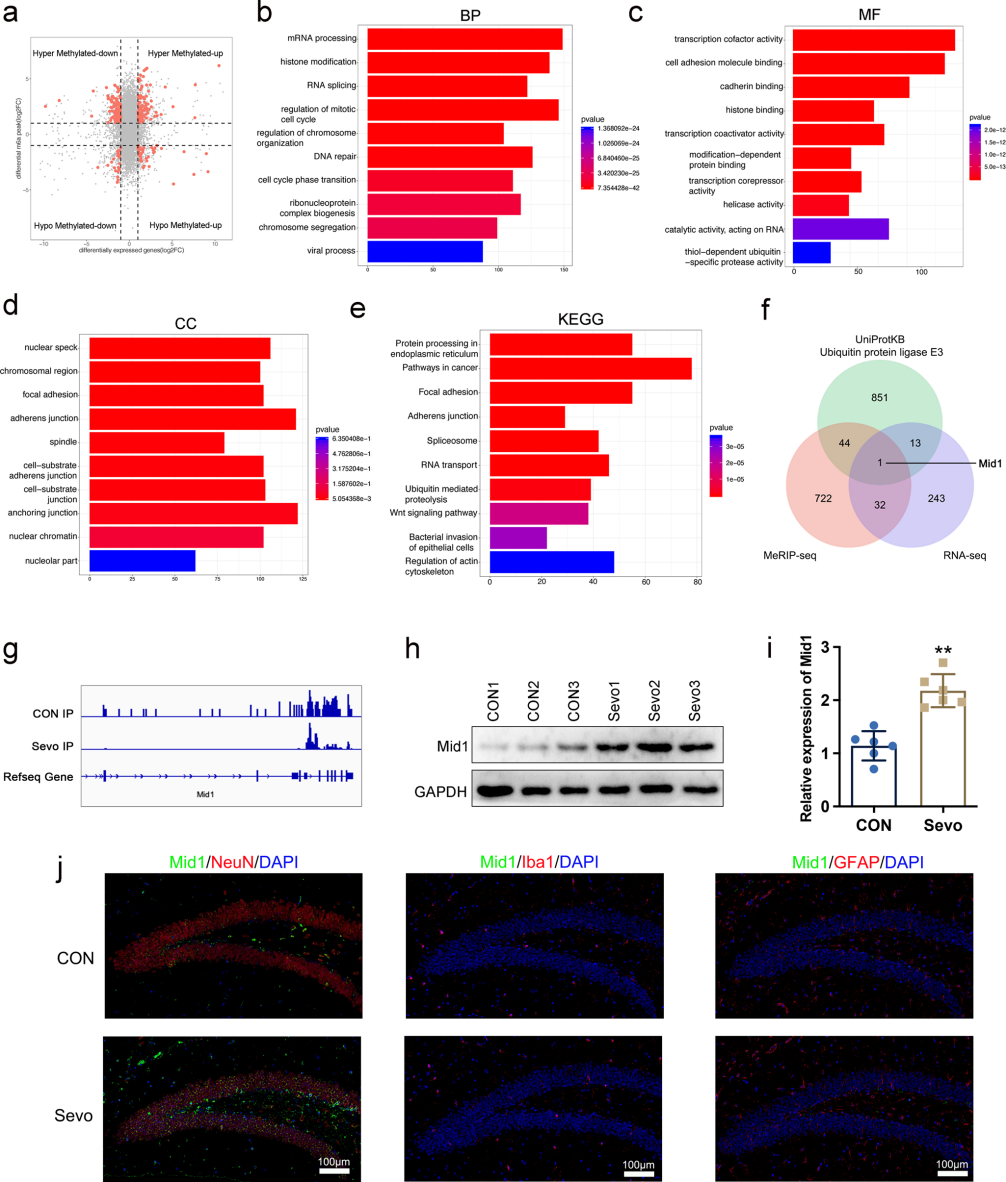
Integrated analysis of m^6^A sequencing and RNA sequencing data reveals a strong correlation between *Mid1* and sevoflurane-induced cognitive impairment. Four-quadrant diagram categorizing 253 differentially methylated m^6^A peaks based on RNA expression levels (part **a**). Gene Ontology analysis results indicating enriched biological processes, molecular functions and cellular components among differentially expressed genes (parts **b**–**d**). Kyoto Encyclopedia of Genes and Genomes (KEGG) pathway analysis illustrating pathways enriched in differentially expressed genes, including protein processing and ubiquitin-mediated proteolysis (part **e**). Venn diagram identifying *Mid1* as a potential target gene through analysis of ubiquitin ligases (part **f**). Visualization of m^6^A methylation levels in *Mid1* mRNA, showing a decrease in the sevoflurane (Sevo) group (part **g**). Western blot analysis and quantitative reverse transcription PCR were used to validate the expression levels of Mid1 after exposure to sevoflurane (parts **h** and **i**). Co-immunofluorescence staining of hippocampal sections for Mid1 with NeuN (neurons), Iba1 (microglia) and GFAP (astrocytes) to determine cell-type specificity of *Mid1* upregulation following sevoflurane exposure (part **j**). CON, control; IP, immunoprecipitation; MeRIP-seq, methylated RNA immunoprecipitation sequencing; RNA-seq, RNA sequencing.

To further explore the target genes of m^6^A modifications, a combined analysis of MeRIP-seq and RNA-seq data was conducted, categorizing the 253 differentially methylated m^6^A peaks with differential RNA levels into four groups. Notably, 194 hypermethylated m^6^A peaks were significantly upregulated (92; hyper-up) or downregulated (102; hyper-down) in RNA, while 59 hypomethylated m^6^A peaks exhibited significant upregulation (39; hypo-up) or downregulation (20; hypo-down) (Fig. 2a). Gene Ontology and Kyoto Encyclopedia of Genes and Genomes (KEGG) pathway analyses were performed on genes with substantial differences in m^6^A peaks and synchronized differential expression to elucidate their biological significance. Gene Ontology analysis revealed that these genes were predominantly enriched in biological processes related to mRNA processing and histone modification (BP), thiol-dependent ubiquitin and cadherin binding (MF), as well as adherens junctions and chromosomal regions (CC) (Fig. 2b–d). KEGG pathway analysis indicated that these genes were primarily enriched in the protein processing pathways in the endoplasmic reticulum and ubiquitin-mediated proteolysis (Fig. 2e). To identify potential ubiquitin ligase E3 candidates associated with these differential genes, Venn diagram analysis was performed using the UniProtKB database, which highlighted *Mid1* as a potential target gene (Fig. 2f). Further visualization using IGV software showed a marked decrease in m^6^A methylation of *Mid1* mRNA in the Sevo group (Fig. 2g). To ascertain the correlation between *Mid1* and cognitive impairment induced by sevoflurane exposure, the expression levels of *Mid1* post-exposure were analysed using western blot analysis and RT-qPCR, revealing a significant upregulation of *Mid1* (Fig. 2h,i). To determine the cell-type specificity of *Mid1* expression in the hippocampus, immunofluorescence staining was performed to examine Mid1 localization in neurons (NeuN⁺), microglia (Iba1⁺) and astrocytes (GFAP⁺); notably, Mid1 was selectively upregulated in NeuN⁺ neurons in the Sevo group, with no obvious increase observed in Iba1⁺ microglia or GFAP⁺ astrocytes (Fig. 2j). These findings suggest that *Mid1* may play a critical role in mediating the cognitive deficits associated with sevoflurane exposure.

### Inhibition of *Mid1* improves cognitive dysfunction induced by sevoflurane exposure

**Fig. 3:**
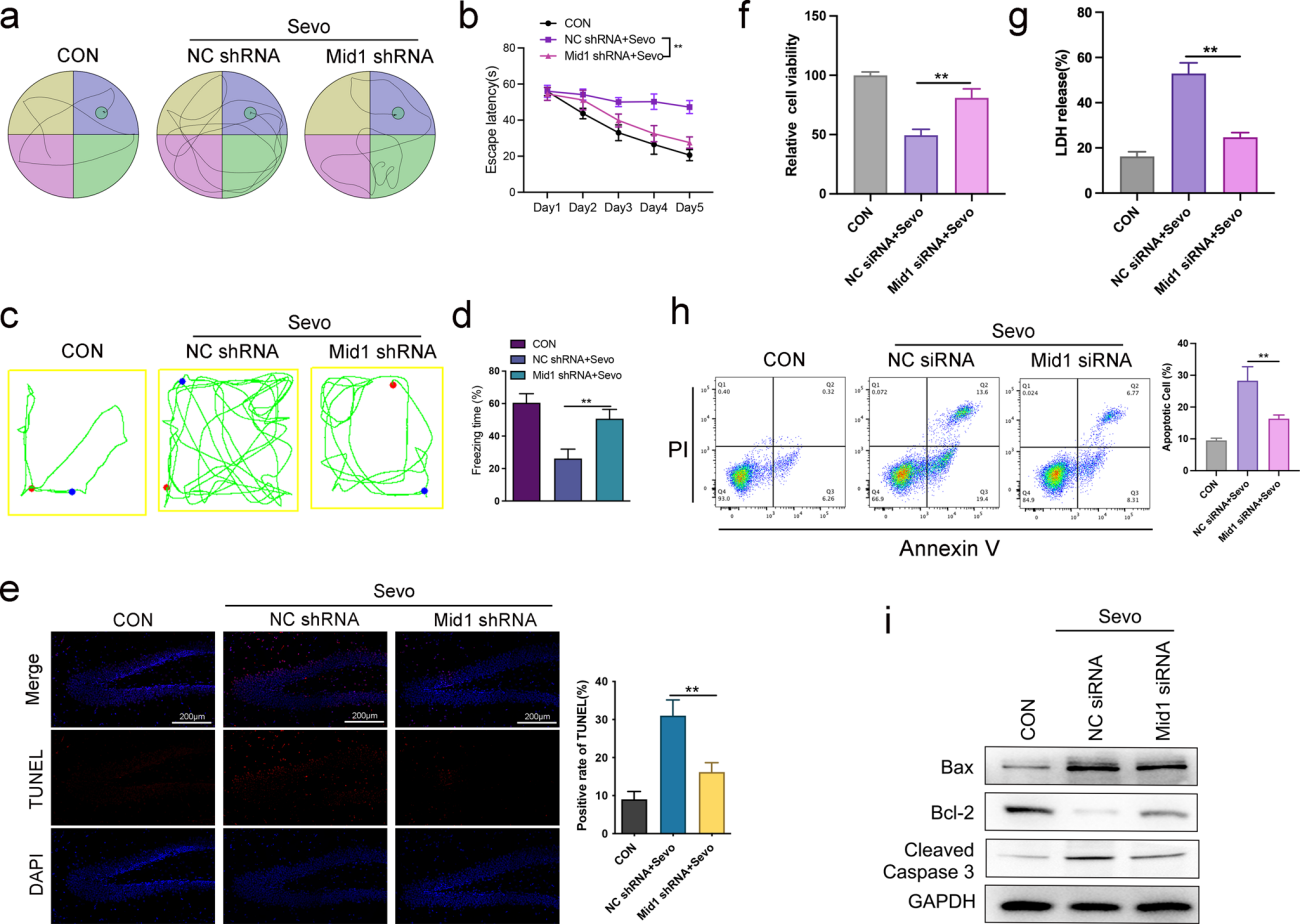
Inhibition of *Mid1* ameliorates cognitive dysfunction induced by sevoflurane exposure. Morris water maze results demonstrating reduced latency to reach the platform following *Mid1* knockdown (parts **a** and **b**). Conditioned fear test data indicating improved freezing behaviour in *Mid1* knockdown mice (parts **c** and **d**). TUNEL assay images showing reduced apoptosis in hippocampal neurons with *Mid1* knockdown (part **e**). The Cell Counting Kit-8 assay was used to validate the effect of Mid1 knockdown on the proliferation of neuronal cells in the presence of sevoflurane (sevo; part **f**). The lactate dehydrogenase (LDH) assay was conducted to assess the toxic effects of *Mid1* knockdown on neuronal cells in the presence of sevoflurane (part **g**). Flow cytometry and western blot analysis were employed to validate the effect of *Mid1* knockdown on the apoptosis of neuronal cells in the presence of sevoflurane (parts **h** and **i**). CON, control; NC, non-coding; PI, propidium iodide; shRNA, short hairpin RNA; siRNA, small interfering RNA.

Various behavioural and histological assessments were employed to evaluate the role of *Mid1* in ameliorating cognitive dysfunction in young mice exposed to sevoflurane. Results from the water maze experiment demonstrated that knockdown of *Mid1* significantly alleviated the prolonged latency period (Fig. 3a,b). Similarly, in the conditioned fear test, *Mid1* knockdown mice exhibited improved freezing behaviour, indicating a restoration of memory and anxiety responses impaired by sevoflurane (Fig. 3c,d). Histological analyses further supported these behavioural findings; TUNEL assays revealed that *Mid1* knockdown effectively reduced the sevoflurane-induced apoptosis of hippocampal neurons (Fig. 3e). In addition, in vitro experiments were conducted to validate the impact of *Mid1* on sevoflurane-induced neurotoxicity. Primary neuronal cells were transfected with non-coding siRNA and *Mid1* siRNA and subsequently subjected to sevoflurane exposure. CCK8 assays indicated a significant increase in cell viability in the *Mid1* siRNA group compared with the non-coding siRNA group, suggesting that *Mid1* knockdown mitigated the cytotoxic effects of sevoflurane (Fig. 3f). LDH-release assays demonstrated that LDH levels in the *Mid1* siRNA group post-sevoflurane treatment were significantly lower than those in the non-coding siRNA group (Fig. 3g). Flow cytometry analysis revealed a marked reduction in apoptosis levels in the *Mid1* siRNA group following sevoflurane treatment (Fig. 3h). Western blot analysis showed that the levels of Bax and cleaved caspase 3 proteins were significantly decreased, while Bcl-2 levels were notably increased in the *Mid1* siRNA group after sevoflurane treatment (Fig. 3i). These findings collectively suggest that *Mid1* knockdown significantly alleviates sevoflurane-induced neurotoxicity, underscoring its pivotal role in preventing cognitive deficits and neuronal damage.

### ALKBH5 and YTHDF2 regulate *Mid1* expression through m^6^A modifications and influence sevoflurane-induced cognitive impairment

**Fig. 4:**
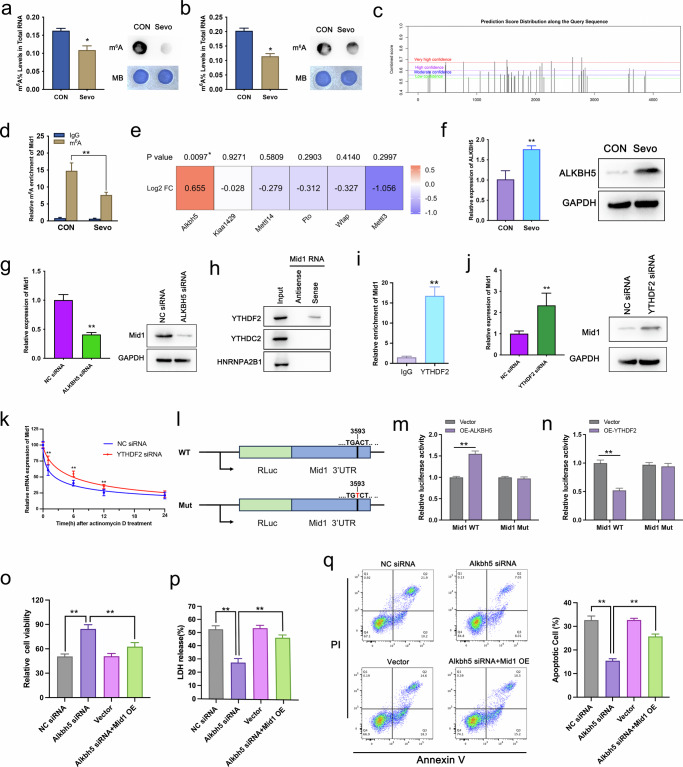
ALKBH5 and YTHDF2 regulate *Mid1* expression through m^6^A modifications. **a**, Overall m^6^A levels in hippocampal tissues from control (CON) and sevoflurane (Sevo) groups. **b**, Similar results for primary neuronal cells, indicating decreased m^6^A levels after sevoflurane exposure. **c**, Identification of high-confidence m^6^A modification sites within the *Mid1* mRNA sequence. **d**, Methylated RNA immunoprecipitation PCR analysis confirming *Mid1* enrichment, with lower levels in the Sevo group. **e**, Expression levels of m^6^A-related enzymes, highlighting upregulation of *Alkbh5* after sevoflurane exposure. **f**, Validation of increased ALKBH5 levels post-exposure using quantitative reverse transcription PCR and western blot analysis. **g**, Impact of *Alkbh5* knockdown on *Mid1* expression. **h**, RNA pull-down assays were performed to analyse the binding of Mid1 to reader proteins. **i**, RIP-qPCR was conducted to validate the interaction between YTHDF2 and Mid1. **j**, The impact of YTHDF2 knockdown on *Mid1* expression was assessed. **k**, Actinomycin D experiments were carried out to examine the stability of *Mid1* mRNA following YTHDF2 knockdown. **l**, Online prediction of candidate m^6^A motifs in the *Mid1* 3′ untranslated region (UTR) using SRAMP; luciferase reporter constructs bearing the mouse *Mid1* 3′ UTR with intact m^6^A motifs or site-directed A to T mutations. **m**, Relative luciferase activity in HEK293T cells co-transfected with plasmids carrying wild-type (WT) or mutant (Mut) *Mid1* 3′ UTR and either OE-ALKBH5 or control vector. **n**, Relative luciferase activity in HEK293T cells co-transfected with plasmids carrying WT or mutant *Mid1* 3′ UTR and either OE-YTHDF2 or control vector. **o**, The Cell Counting Kit-8 assay was used to evaluate the proliferation of neuronal cells in rescue experiments involving Alkbh5 knockdown and *Mid1* overexpression. **p**, The lactate dehydrogenase (LDH) assay was performed to assess the toxic effects on neuronal cells in rescue experiments involving *Alkbh5* knockdown and *Mid1* overexpression. **q**, Flow cytometry was used to detect apoptosis in neuronal cells during rescue experiments involving *Alkbh5* knockdown and *Mid1* overexpression. NC, non-coding; PI, propidium iodide; siRNA, small interfering RNA.

The overall m^6^A levels in hippocampal tissue before and after sevoflurane exposure were compared, with quantitative analysis and dot blot results indicating a significant reduction in overall m^6^A levels in the Sevo group (Fig. 4a). A similar trend was observed in primary neuronal cells (Fig. 4b). Using the SRAMP tool (http://www.Cuilab.cn/sramp), the m^6^A modification sites within the *Mid1* mRNA sequence were analysed and several high-confidence m^6^A modification sites were identified (Fig. 4c). MeRIP-PCR analysis was conducted to further validate the m^6^A modifications of *Mid1*, demonstrating significant enrichment of *Mid1* in both the CON and Sevo groups, with lower levels observed in the Sevo group than in the CON group (Fig. 4d). m^6^A is a reversible RNA modification, regulated by m^6^A methyltransferases and demethylases. Analysis of m^6^A-related enzyme expression in RNA-seq data before and after sevoflurane exposure revealed a significant upregulation of the demethylase ALKBH5 (*P* = 0.0097) (Fig. 4e). RT-qPCR and western blot analyses confirmed that ALKBH5 levels were significantly elevated following sevoflurane exposure (Fig. 4f). Notably, siRNA-mediated knockdown of *Alkbh5* in neuronal cells led to a significant decrease in *Mid1* expression (Fig. 4g). Thus, it can be concluded that the m^6^A modification of *Mid1* is regulated by ALKBH5. Previous studies have shown that, when m^6^A in mRNAs is recognized by reader proteins such as YTHDF, YTHDC and HNRNPA2B1, it can promote mRNA decay or translation. In the present experiments, RNA pull-down assays revealed that Mid1 could significantly bind to the reader protein YTHDF2 (Fig. 4h). RNA immunoprecipitation followed by qPCR (RIP-qPCR) further confirmed the interaction between YTHDF2 and Mid1, showing significant enrichment of *Mid1* RNA in neuronal cells compared with the control IgG group (Fig. 4i). To determine whether YTHDF2 affects *Mid1* expression, YTHDF2 in neuronal cells were knocked down, resulting in upregulation of *Mid1* at both RNA and protein levels (Fig. 4j). In addition, the impact of YTHDF2 on the stability of *Mid1* RNA was analysed. Following transfection with YTHDF2 siRNA and non-coding siRNA, total RNA was harvested at various time points after treatment with actinomycin D. The absence of YTHDF2 significantly enhanced the stability of *Mid1* RNA (Fig. 4k). In summary, ALKBH5 and YTHDF2 regulate *Mid1* expression through m^6^A modifications, highlighting their critical roles in cognitive impairment induced by sevoflurane. To determine whether ALKBH5 regulates *Mid1* expression via m⁶A modification, the *Mid1* mRNA sequence was analysed and the SRAMP predictor was used to identify DRACH motifs within the 3′ UTR (D = A/G/U; R = purine; H = A/C/U). Either the wild-type *Mid1* 3′ UTR or an m⁶A-deficient 3′ UTR (A to T substitutions at the predicted motifs) was then cloned into dual-luciferase reporters (Fig. 4l) and the reporter activity in transfected HEK293T cells was measured. *Alkbh5* overexpression led to a significant increase in luciferase activity for the wild-type 3′ UTR reporter, whereas the mutant 3′ UTR reporter showed no appreciable change (Fig. 4m). Conversely, YTHDF2 overexpression reduced the wild-type reporter signal but had minimal effect on the mutant (Fig. 4n). These findings indicate that intact m⁶A sites are required for ALKBH5-mediated stabilization, consistent with a YTHDF2-dependent post-transcriptional decay mechanism.

Furthermore, the effect of *Alkbh5*-mediated regulation of *Mid1* expression on sevoflurane-induced neurotoxicity in vitro was validated. *Alkbh5* siRNA was transfected while simultaneously overexpressing *Mid1* in primary neuronal cells for rescue experiments, followed by sevoflurane exposure. CCK8 assays indicated that cell viability in the *Alkbh5* siRNA group was significantly higher than in the non-coding siRNA group. However, cell viability in the *Alkbh5* siRNA group combined with *Mid1* overexpression was significantly reduced compared with the *Alkbh5* siRNA group alone, indicating that *Mid1* overexpression reversed the alleviation of sevoflurane-induced cytotoxicity observed with *Alkbh5* knockdown (Fig. 4o). LDH-release assays revealed that LDH levels in the *Alkbh5* siRNA group following sevoflurane treatment were significantly lower than those in the non-coding siRNA group, while LDH levels in the *Alkbh5* siRNA group combined with *Mid1* overexpression were significantly elevated compared with the *Alkbh5* siRNA group (Fig. 4p). Flow cytometry analysis showed that the *Alkbh5* siRNA group significantly reduced apoptosis levels following sevoflurane treatment, while *Alkbh5* siRNA combined with *Mid1* overexpression reversed this effect (Fig. 4q). Overall, m^6^A modifications regulating *Mid1* expression play a crucial role in sevoflurane-induced cognitive impairment.

### Mid1 regulates the ubiquitination process of Syngap1

**Fig. 5:**
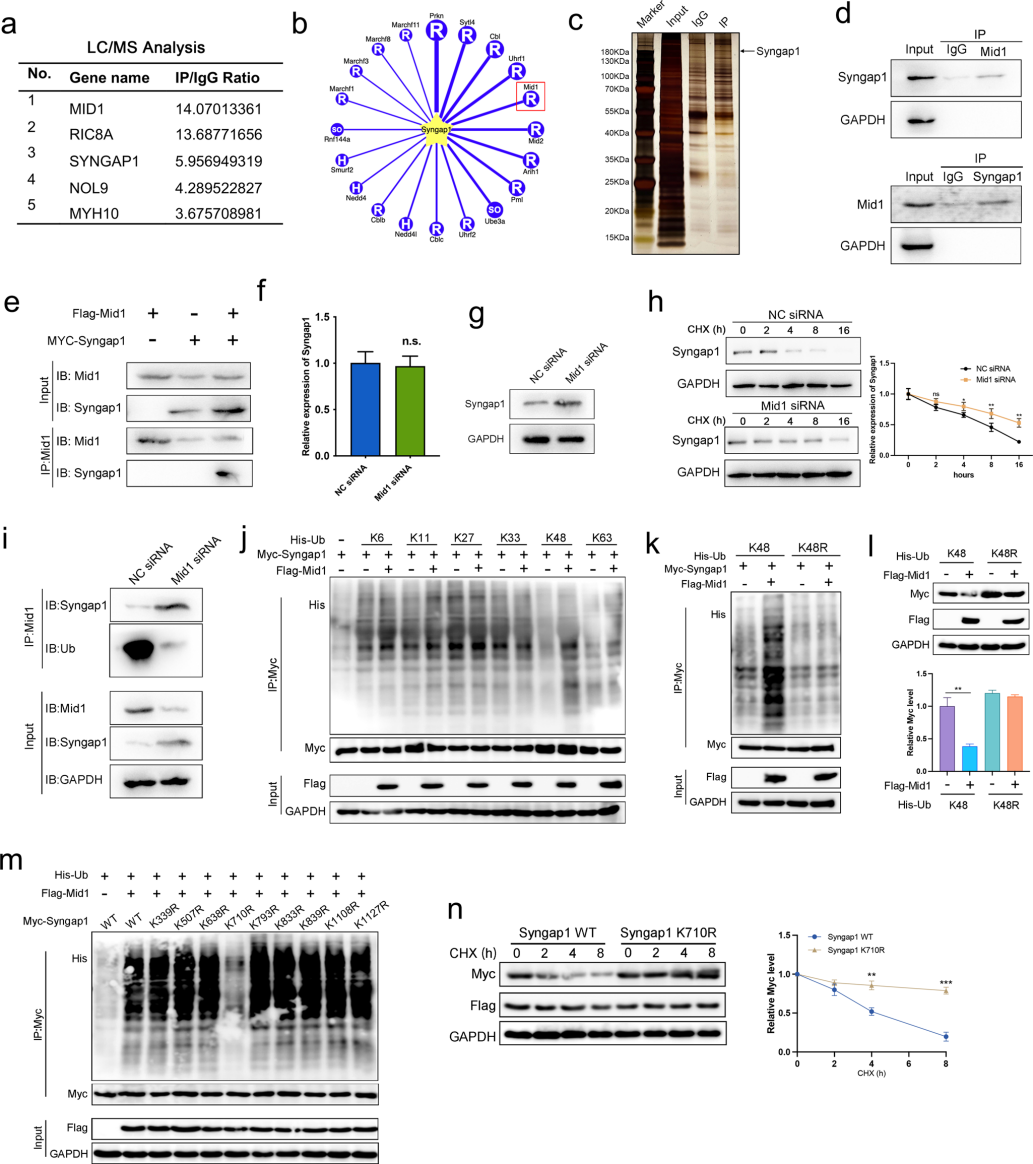
Mid1 regulates the ubiquitination process of Syngap1. Identification of proteins interacting with Mid1 using mass spectrometry (parts **a** and **b**). Silver staining showing specific bands for Syngap1 in the Mid1 immunoprecipitation group (part **c**). Co-immunoprecipitation (Co-IP) assay confirming Mid1 and Syngap1 interaction in neuronal cells (part **d**). Validation of interaction in HEK293T cells co-transfected with Flag-Mid1 and Syngap1 (part **e**). Analysis of Syngap1 protein levels following *Mid1* knockdown (parts **f** and **g**). Western blot analysis showing Syngap1 half-life in *Mid1* knockdown cells after cycloheximide (CHX) treatment (part **h**). Levels of ubiquitinated Syngap1 following Mid1 knockdown (part **i**). HEK293T cells co-transfected with Flag-Mid1, Myc-Syngap1 and the indicated His-Ub plasmids; assessment of Myc-Syngap1 ubiquitination (part **j**). Co-IP analysis of HEK293T cells expressing Myc-Syngap1 and His-ubiquitin (K48 or K48R) with or without Flag-Mid1 (part **k**). Lysate analysis of HEK293T cells co-expressing Flag-Mid1 and Myc-Syngap1 and transfected with K48 or K48R Ub; quantification by immunoblotting (part **l**). Co-IP analysis of HEK293T cells transfected with His-Ub, Flag-Mid1, and Myc-Syngap1 or the indicated mutants (part **m**). Immunoblotting of lysates from HEK293T cells co-transfected with Myc-tagged Syngap1 (wild-type (WT) or K710R) and Flag-Mid1, with or without CHX (10 μg/ml), using anti-Flag and anti-Myc antibodies at the indicated time points (part **n**). NC, non-coding; siRNA, small interfering RNA.

To elucidate the molecular mechanisms by which *Mid1* contributes to sevoflurane-induced cognitive impairment, proteins interacting with Mid1 were identified through mass spectrometry and immunoprecipitation analysis, listing the top five differential proteins. Using the UbiBrowser database (http://ubibrowser.bio-it.cn/ubibrowser/), it was validated that differential proteins potentially bind to ubiquitin E3 ligases, with Syngap1 predicted as a substrate of Mid1 (Fig. 5a,b). Silver staining revealed a specific protein band for Syngap1 in the Mid1 immunoprecipitation group compared with the IgG group (Fig. 5c). To further explore the interaction between Mid1 and Syngap1, Co-IP assays were performed using anti-Mid1 and anti-Syngap1 antibodies in neuronal cells, confirming their endogenous interaction (Fig. 5d). In addition, Co-IP assays conducted in HEK293T cells co-transfected with Flag-Mid1 and Syngap1 plasmids further validated their interaction (Fig. 5e). Notably, these experiments demonstrated that knockdown of Mid1 expression in neuronal cells did not affect *Syngap1* mRNA levels but led to a significant increase in Syngap1 protein levels (Fig. 5f,g), indicating a post-transcriptional regulatory mechanism. To elucidate the regulatory mechanism by which Mid1 influences Syngap1 stability, CHX treatment was employed to block protein translation; western blot analysis showed a significantly prolonged half-life of Syngap1 in *Mid1* knockdown cells (Fig. 5h). Furthermore, a marked downregulation of ubiquitinated Syngap1 levels following *Mid1* knockdown was observed (Fig. 5i). To define the chain type responsive to Mid1-mediated ubiquitination of Syngap1, Mid1, Syngap1 and chain-restricted ubiquitin variants (K6/K11/K29/K33/K48/K63) were co-expressedin HEK293T cells. Only K48-linked polyubiquitination was markedly increased in the presence of Mid1 (Fig. 5j). Consistently, transfection with the ubiquitin K48R mutant significantly abolished both Mid1 overexpression-induced downregulation of Syngap1 protein and the increase in Syngap1 ubiquitination (Fig. 5k,l). To further pinpoint the Syngap1 ubiquitination site responsible for Mid1-induced modification, the iPTMnet database was used to design nine Syngap1 mutants in which lysine (K) residues were substituted with arginine (R), and each mutant was co-transfected with Flag-Mid1 and His-tagged K48-only ubiquitin into HEK293T cells. Only the K710R mutant was found to significantly abrogate the ubiquitination level (Fig. 5m). Under protein-synthesis blockade (CHX treatment), Mid1 no longer reduced K710R protein levels (Fig. 5n), supporting K710 as a functional acceptor lysine. Collectively, these findings underscore the pivotal role of Mid1 in regulating Syngap1 stability through ubiquitin-mediated degradation, revealing a potential mechanism of neurotoxicity in the neuronal environment.

### *Mid1* mediates the MAPK signalling pathway through regulation of Syngap1 in sevoflurane-induced cognitive impairment

**Fig. 6:**
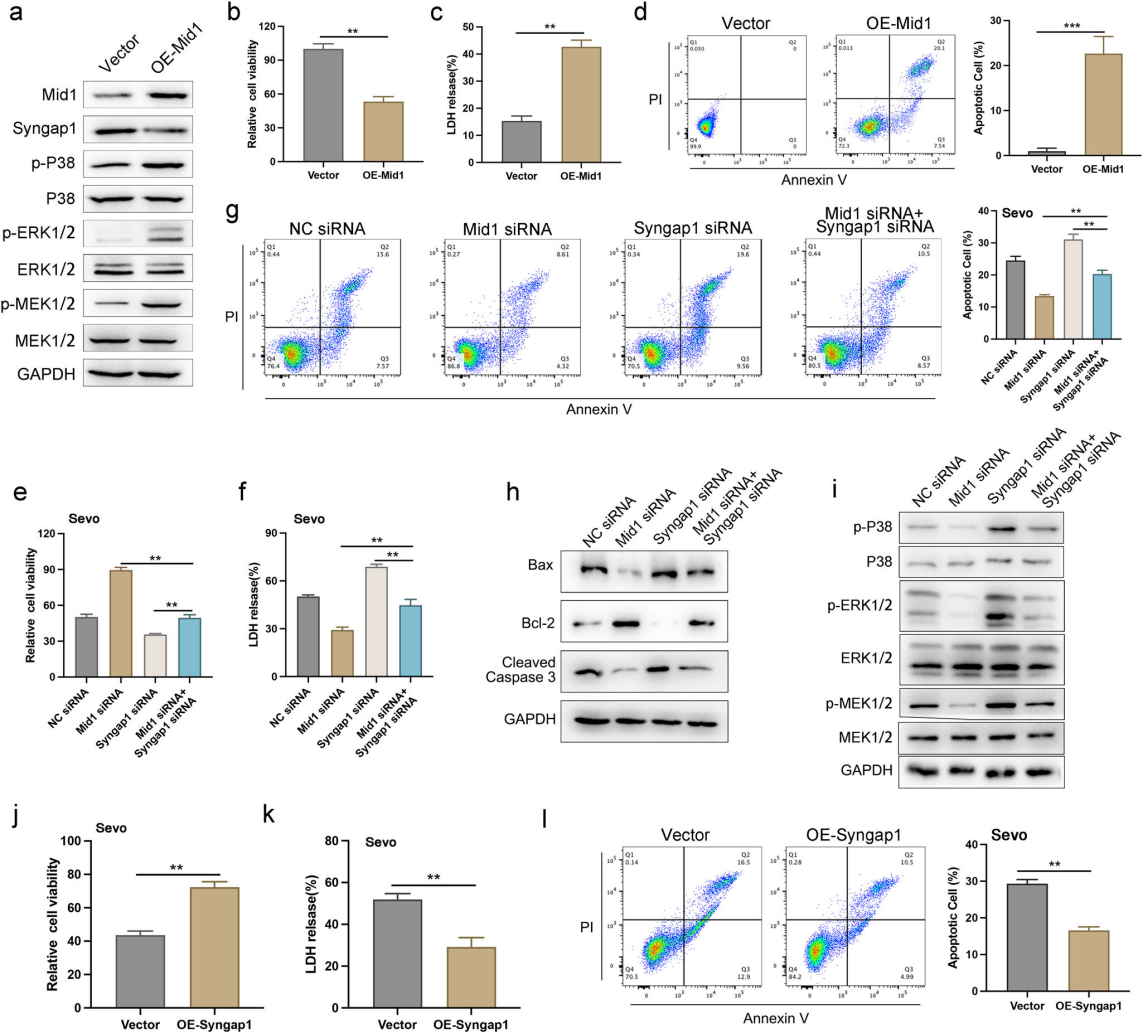
*Mid1* mediates the MAPK signalling pathway through regulation of Syngap1. Western blot analysis of Syngap1 protein and MAPK pathway activation markers in neurons overexpressing *Mid1* (part **a**). Cell Counting Kit-8 (CCK8) assay results demonstrating cell viability in neurons overexpressing *Mid1* (part **b**). Lactate dehydrogenase (LDH) release assay quantifying cell death of neurons overexpressing *Mid1* (part **c**). Apoptosis quantified by an apoptosis assay (part **d**). CCK8 assay results demonstrating cell viability in co-transfected neuronal cells after sevoflurane (Sevo) exposure (part **e**). LDH-release assay quantifying cell death in response to Sevo treatment (part **f**). Flow cytometry and western blot analysis showing apoptosis levels in neuronal cultures (parts **g** and **h**). Western blot analysis of the activation status of the MAPK signalling pathway (part **i**). CCK8 assay showing cell viability in Syngap1-overexpressing neurons after Sevo exposure (part **j**). LDH-release assay quantifying cell death in Syngap1-overexpressing neurons in response to sevoflurane (part **k**). Flow cytometry measuring apoptosis in neuronal cultures overexpressing Syngap1 after Sevo treatment (part **l**). NC, non-coding; PI, propidium iodide; siRNA, small interfering RNA.

To directly assess the sufficiency of *Mid1*, *Mid1* was overexpressed in primary neurons under sevoflurane-free conditions. *Mid1* overexpression markedly decreased Syngap1 protein levels and concomitantly activated the MAPK pathway (Fig. 6a). Reduced cell viability, elevated LDH release and increased apoptosis were also observed (Fig. 6b–d). To investigate the role of *Mid1* in regulating Syngap1 and its implications in sevoflurane-induced neurotoxicity, rescue experiments were conducted by transfecting neuronal cells with *Mid1* siRNA, *Syngap1* siRNA and a combination of both, followed by sevoflurane exposure. CCK8 assays indicated that cell viability in the co-transfection group was significantly lower than viability in the *Mid1* siRNA group, while the co-transfection group exhibited significantly higher cell viability than the *Syngap1* siRNA group, suggesting that *Syngap1* knockdown partially reversed the cytotoxic effects of sevoflurane seen with *Mid1* knockdown (Fig. 6e). LDH-release assays demonstrated that LDH levels in the co-transfection group were significantly elevated compared with levels in the *Mid1* siRNA group, while the levels were significantly reduced compared with those in the *Syngap1* siRNA group (Fig. 6f). Flow cytometry analysis revealed that apoptosis levels in the co-transfection group were significantly higher than levels in the *Mid1* siRNA group, while the co-transfection group exhibited significantly lower cell apoptosis than the *Syngap1* siRNA group (Fig. 6g). Western blot analysis indicated that the levels of Bax and cleaved caspase 3 proteins were significantly elevated in the co-transfection group compared with the *Mid1* siRNA group, while Bcl-2 levels were notably decreased; conversely, the co-transfection group exhibited decreased levels of Bax and cleaved caspase 3 proteins and increased Bcl-2 levels compared with the *Syngap1* siRNA group (Fig. 6h). Compared with the non-coding siRNA group, phosphorylation levels of P38, ERK1/2 and MEK1/2 were significantly reduced in the *Mid1* siRNA group, while phosphorylation levels of P38, ERK1/2 and MEK1/2 were significantly increased in the *Syngap1* siRNA group. In contrast to the *Mid1* siRNA group, the co-transfection group reversed the downregulation of phosphorylation levels of P38, ERK1/2 and MEK1/2; conversely, compared with the *Syngap1* siRNA group, the co-transfection group reversed the increase in phosphorylation levels of P38, ERK1/2 and MEK1/2 (Fig. 6i). Additionally, complementary gain-of-function experiments were performed by overexpressing Syngap1. In primary neurons exposed to sevoflurane, Syngap1 overexpression significantly attenuated the injury phenotype, restoring cell proliferation, lowering LDH release and reducing apoptosis (Fig. 6j–l). These findings suggest that *Mid1* regulates Syngap1, which mediates the MAPK signalling pathway, playing a crucial role in the cognitive impairment induced by sevoflurane.

### In vivo study of the role of *Mid1* in regulating Syngap1 in sevoflurane-induced cognitive impairment

**Fig. 7:**
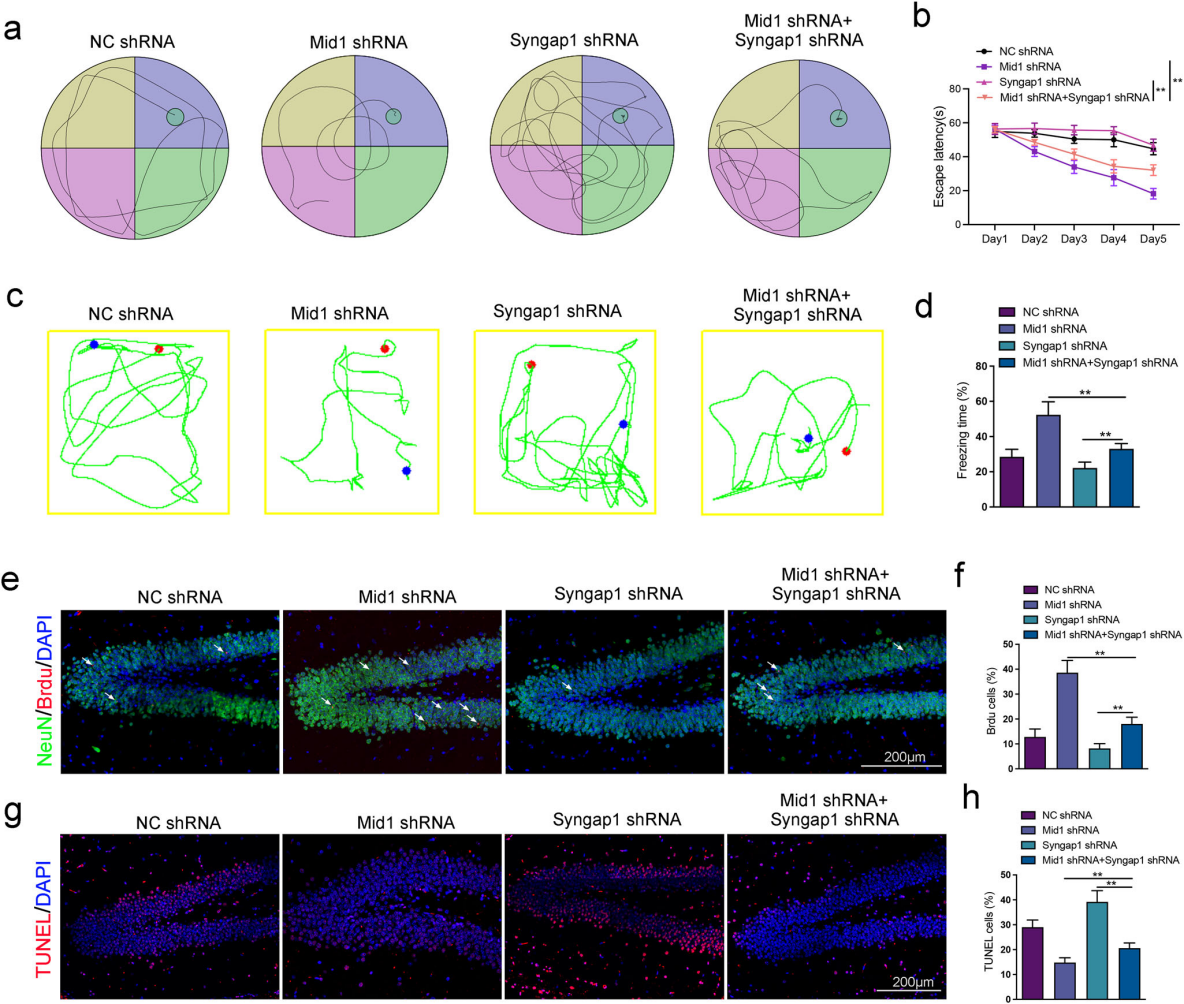
In vivo investigation of the role of *Mid1* in regulating Syngap1 in sevoflurane-induced cognitive impairment. The water maze test showed changes in escape latency (parts **a** and **b**). The contextual fear conditioning test revealed variations in freezing time (parts **c** and **d**). Histological analysis of BrdU in brain sections was used to assess the proliferation of hippocampal neurons (parts **e** and **f**). Histological analysis of TUNEL staining in brain sections evaluated the apoptosis of hippocampal neurons (parts **g** and **h**). NC, non-coding; shRNA, short hairpin RNA.

To determine whether *Mid1* mediates sevoflurane-induced neurotoxicity through the regulation of Syngap1 in vivo, P2 neonatal C57BL/6 mice were intracranially injected with non-coding shRNA, *Mid1* shRNA, *Syngap1* shRNA, and a combination of *Mid1* shRNA and *Syngap1* shRNA. Subsequently, the neuroprotective effects of Mid1 and Syngap1 in the sevoflurane-exposed mouse model were assessed through behavioural and histological evaluations. The results from the water maze experiment indicated that the combination group showed a reversal in the latency decrease induced by *Mid1* shRNA and the latency increase caused by *Syngap1* shRNA (Fig. 7a,b). Similarly, in the conditioned fear test, the combination group showed a reversal in the increased freezing behaviour induced by *Mid1* shRNA and the decreased freezing behaviour caused by *Syngap1* shRNA (Fig. 7c,d). Histological analyses further demonstrated that BrdU proliferation staining indicated a reversal of the increased proliferation caused by *Mid1* shRNA and the decreased proliferation induced by *Syngap1* shRNA in the combination group (Fig. 7e,f). In addition, TUNEL assays revealed that the combination group showed a reversal in the reduction in hippocampal neuronal apoptosis induced by *Mid1* shRNA and the increase in apoptosis caused by *Syngap1* shRNA (Fig. 7g,h). These results provide compelling evidence that *Mid1* promotes neurotoxicity and cognitive impairment following sevoflurane exposure through the regulation of Syngap1.

## Discussion

Research into the cognitive effects of sevoflurane exposure, particularly during the early developmental stages in young individuals, is increasingly relevant due to the implications for long-term neurodevelopmental outcomes^20–22^. Cognitive impairments associated with anaesthetic exposure during critical periods of brain development can lead to significant deficits in learning and memory, which have been documented in various animal models^23,24^. For instance, studies have shown that sevoflurane can induce neuroinflammation and neuronal apoptosis in neonatal rodents, subsequently affecting their cognitive functions later in life^25–27^. Furthermore, the mechanisms underlying these impairments, including the roles of protein modifications such as m^6^A methylation and ubiquitination, are not fully understood but are critical for elucidating the pathways that contribute to anaesthetic-induced neurotoxicity^10,28–31^.

In this study, the molecular mechanisms linking sevoflurane exposure to cognitive deficits in neonatal mice were investigated, particularly focusing on the roles of m^6^A modifications and the ubiquitin–proteasome system. These findings provide insights into how the regulation of key genes, such as *Mid1* and *Syngap1*, intersects with cognitive function and synaptic integrity during anaesthetic exposure^32^. The research highlights the importance of understanding these molecular pathways as they may pave the way for the development of targeted therapeutic strategies to mitigate cognitive decline associated with surgical anaesthetics^33^.

Investigating the cognitive effects of sevoflurane exposure in neonatal mice reveals significant insights into the molecular mechanisms and signalling pathways involved in anaesthetic-induced neurotoxicity. This study demonstrates that *Mid1*, a critical E3 ubiquitin ligase, plays a pivotal role in regulating the stability of Syngap1, a protein integral to synaptic plasticity. The findings indicate that the knockdown of *Mid1* leads to increased levels of Syngap1 protein without altering its mRNA expression, suggesting that the regulation occurs at a post-transcriptional level through ubiquitin-mediated degradation^13,34–36^. This discovery elucidates a previously unrecognized mechanism whereby *Mid1* influences cognitive outcomes following anaesthetic exposure, thereby highlighting its potential as a therapeutic target for the prevention of cognitive decline associated with sevoflurane anaesthesia. Such insights could inform the development of strategies aimed at mitigating the long-term neurodevelopmental consequences of anaesthetics, reinforcing the significance of understanding these molecular interactions in the context of paediatric anaesthesia.

Furthermore, these results reveal the involvement of the MAPK signalling pathway in cognitive impairment induced by sevoflurane^37^. The regulation of Syngap1 by *Mid1* impacts the phosphorylation levels of key components within the MAPK pathway, including p38 and MEK1/2. Understanding this signalling axis is crucial, as it identifies critical nodes for potential pharmacological intervention that could reverse cognitive deficits following anaesthetic exposure. Targeting this pathway may provide new avenues for therapeutic development, offering hope for children at risk of neurodevelopmental disorders due to anaesthetic exposure. Therefore, these findings not only contribute to the existing body of knowledge regarding anaesthetic neurotoxicity but also pave the way for innovative strategies aimed at safeguarding cognitive function during critical periods of brain development.

In addition to elucidating the molecular mechanisms, this study highlights the functional implications of *Mid1* and Syngap1 on cognitive behaviour in sevoflurane-exposed mice. Behavioural assessments indicated that *Mid1* knockdown led to significant improvements in cognitive functions, as demonstrated by enhanced performance in the Morris water maze and conditioned fear tests. These results correlate molecular mechanisms with observable behaviours, underscoring the relevance of *Mid1* in cognitive health. The demonstrated potential for behavioural interventions targeting cognitive impairments offers promise for clinical applications, emphasizing the importance of further research into the relationship between molecular targets and cognitive outcomes. This work contributes to a greater understanding of the intricate interplay between anaesthetic exposure, cognitive function and the underlying molecular pathways, ultimately aiming to improve anaesthetic safety in paediatric populations.

The limitations of this study should be acknowledged to contextualize the findings within the broader scope of cognitive impairment research. First, while the use of an animal model provides valuable insights, the translational relevance to humans remains uncertain due to inherent biological differences. In addition, the sample size utilized in the experiments may limit the generalizability of the results, warranting further validation through larger-scale studies. Furthermore, the absence of longitudinal assessments restricts understanding of the long-term cognitive outcomes following sevoflurane exposure, leaving potential chronic effects unexplored. Finally, although the molecular mechanisms involving *Mid1* and Syngap1 have been elucidated, the complexity of neurodevelopmental processes requires caution when extrapolating these findings to multifactorial conditions such as paediatric anaesthesia neurotoxicity.

In conclusion, this research highlights the critical role of *Mid1* in mediating the cognitive dysfunction associated with sevoflurane exposure through mechanisms involving m^6^A modifications and the regulation of Syngap1. The findings provide a framework for understanding the molecular underpinnings of anaesthetic-induced neurotoxicity, suggesting potential avenues for therapeutic interventions aimed at mitigating cognitive deficits. By elucidating the interplay between m^6^A modifications and ubiquitination in neurodevelopment, this study paves the way for future investigations that may lead to novel strategies to enhance anaesthetic safety in paediatric populations, ultimately contributing to improved long-term cognitive health outcomes.

## Data Availability

The authors declare that all data supporting the findings of this study are available within the article.
